# Resource use, costs and quality of end-of-life care: observations in a cohort of elderly Australian cancer decedents

**DOI:** 10.1186/s13012-014-0148-2

**Published:** 2015-02-26

**Authors:** Julia M Langton, Preeyaporn Srasuebkul, Rebecca Reeve, Bonny Parkinson, Yuanyuan Gu, Nicholas A Buckley, Marion Haas, Rosalie Viney, Sallie-Anne Pearson, The End-of-Life in Cancer Care (EoL-CC) Investigators

**Affiliations:** Faculty of Pharmacy, The University of Sydney, Sydney, NSW Australia; Centre for Health Economics Research & Evaluation, The University of Technology Sydney, Sydney, NSW Australia; Sydney Medical School, The University of Sydney, Sydney, NSW Australia

**Keywords:** End-of-life, Cancer, Palliative care, Resource utilization, Costs, Patterns of care

## Abstract

**Background:**

The last year of life is one of the most resource-intensive periods for people with cancer. Very little population-based research has been conducted on end-of-life cancer care in the Australian health care setting. The objective of this program is to undertake a series of observational studies examining resource use, costs and quality of end-of-life care in a cohort of elderly cancer decedents using linked, routinely collected data.

**Methods/Design:**

This study forms part of an ongoing cancer health services research program. The cohorts for the end-of-life research program comprise Australian Government Department of Veterans’ Affairs decedents with full health care entitlements, residing in NSW for the last 18 months of life and dying between 2005 and 2009. We used cancer and death registry data to identify our decedent cohorts and their causes of death. The study population includes 9,862 decedents with a cancer history and 15,483 decedents without a cancer history. The median age at death is 86 and 87 years in the cancer and non-cancer cohorts, respectively. We will examine resource use and associated costs in the last 6 months of life using linked claims data to report on health service use, hospitalizations, emergency department visits and medicines use. We will use best practice methods to examine the nature and extent of resource use, costs and quality of care based on previously published indicators. We will also examine factors associated with these outcomes.

**Discussion:**

This will be the first Australian research program and among the first internationally to combine routinely collected data from primary care and hospital-based care to examine comprehensively end-of-life care in the elderly. The research program has high translational value, as there is limited evidence about the nature and quality of care in the Australian end-of-life setting.

**Electronic supplementary material:**

The online version of this article (doi:10.1186/s13012-014-0148-2) contains supplementary material, which is available to authorized users.

## Background

Cancer care imposes a significant burden on health systems globally with the year following diagnosis and last year of life being the most resource-intensive stages of care [1]. Relative to the evidence-base supporting clinical decision-making at the time of a cancer diagnosis, there is limited understanding about what constitutes quality end-of-life care [2-5]. Randomized trials of end-of-life treatments and services remain rare and likely to remain so for ethical and practical reasons [6-9]. Therefore, researchers need to utilize other methods and data to examine this important area of medical practice. Observational research on end-of-life care can enhance our understanding of patterns of care in real-world clinical settings and assist in establishing evidence to inform clinical practice, resource allocation and planning decisions.

Observational studies using linked health administrative datasets to explore patterns of end-of-life care have increased in recent years. The use of existing data such as billing claims, linked with cancer and death registry data facilitates the creation of decedent cohorts to obtain a comprehensive picture of treatment patterns [3]. These datasets have the advantage of being less costly and broader in scope than primary data collections that are time- and cost-intensive and generally undertaken in small numbers of highly selected patients and settings.

We recently conducted a systematic review of all observational end-of-life studies using administrative health datasets published over a 20-year period [10]. We synthesized the outcomes of 78 studies that collectively examined end-of-life resource use and/or costs in over 3.7 million cancer decedents. Despite this large volume of published work, some important gaps in knowledge were evident. First, end-of-life care is a global concern, yet little research has been generated outside the North American setting. Second, most of the studies in the review focused on hospital care; few examined the full spectrum of treatments received at life’s end including medications, palliative services, physician visits and community care. Finally, few studies compared resource utilization or costs in cancer patients to those without a cancer diagnosis.

We identified five Australian studies in the systematic review [7-9,11,12] that focused on the use of specialist palliative care services, hospital admissions or emergency department presentations in a specific end-of-life period. All but one study was conducted in the state of Western Australia, and only one included data derived in the last decade. While these studies demonstrate the opportunities to explore patterns of end-of-life care in the Australian setting, they also highlight the need for more contemporary data as well as examining the full range of health care provision including medication use and community-based medical care.

The program of research outlined in this protocol makes use of linked, routinely collected data to examine resource use, costs and quality of end-of-life cancer care. We will also examine the factors associated with these outcomes. We have brought together population-based datasets collected across multiple Australian health care jurisdictions to create decedent cohorts with and without a previous cancer history to explore how end-of-life care varies in different patient populations and according to different causes of death.

## Methods

### Setting

Australia has a publicly funded universal health care system entitling all Australian citizens and permanent residents to a range of subsidized health services. This includes free treatment in public hospitals (funded jointly by the Commonwealth and State/Territory governments) and subsidized treatment in private hospitals (funded jointly by the Commonwealth and private health insurance). It also includes a range of subsidized outpatient services including consultations with medical and selected health care professionals (funded by the Commonwealth’s Medicare Benefits Scheme, MBS) and medicines prescribed in hospitals and the community (funded by the Commonwealth’s Pharmaceutical Benefits Scheme, PBS; medicines prescribed to public hospital inpatients are covered primarily by the hospital budget).

Our end-of-life research program has been developed from a larger cancer health services research program that commenced in 2011. The program focuses on clients of the Australian Government Department of Veterans’ Affairs (DVA). The DVA funds the health care of eligible veterans, war widows and widowers and their dependants. Eligible persons with Gold Repatriation Health Cards (Gold Card Holders) are entitled to treatment for all conditions (i.e. all health services subsidized for Australian citizens plus additional DVA-approved services and pharmaceutical items not subsidized for the general population). White Card Holders are entitled to treatment for specific-conditions approved by the DVA (other conditions will be treated and subsidized according to the general population entitlements). The Orange Repatriation Pharmaceuticals Benefits Card provides eligible British, other Commonwealth or allied veterans subsidized access to approved pharmaceuticals according to clinical need [13].

DVA clients are a major subgroup of the Australian population. In December 2010, they constituted approximately 6% of those aged 65 years and older and 27% of Australians aged at least 85 years [14]. Our research program is limited to all DVA clients residing in NSW (Australia’s largest state by population). DVA clients residing in NSW account for 34% of the Australian DVA population and have a similar age and gender profile to clients residing in other Australian states [14]. Additionally, when compared to Australians of similar age, DVA clients have very similar rates of health service use [15].

### Overview of data sources

The data infrastructure comprises DVA data holdings linked with NSW Ministry of Health data collections. The DVA datasets contain information on services provided to DVA clients regardless of the location in which they occurred in Australia; the NSW collections contain only data on services provided in NSW. Our current data holdings are summarized in Table 1 and detailed below.

**Table 1:** **Current data holdings**

**Dataset**	**Description**	**Data custodian**	Data holdings (at October 2013)*	**Coding/classification**	**Cancer cohorts**	**Fact of death**	**Cause of death**	**Resource use/costs**
Patient information								
Client file	Demographic information and level of entitlement	DVA	April 1992–January 2010	-		X		
Residential aged care	Residence in aged care facility	DVA	July 2005–March 2011	-				
Registries								
Central Cancer Registry	Cancer notifications in NSW	NSW MoH	January 1994–December 2009	ICD-10 ICD-O-3	X	x	X	
Registry of Births, Deaths and Marriages (RBDM)	Deaths in NSW	NSW MoH	January 1994–December 2012	-	x	x	x	
Cause of death (part of RBDM)	Cause of death in NSW (coded by Australian Bureau of Statistics)	NSW MoH	January 1994–December 2007	ICD-9 (94–98) ICD-10-AM (from 97)	x	x	x	
Health service and prescribed medicines datasets^								
Repatriation Pharmaceutical Benefits Scheme	Pharmaceutical items in the Pharmaceutical Benefits Scheme (PBS) and extra items paid for by the DVA, dispensed anywhere in Australia	DVA	July 2004–January 2010	R/PBS items mapped to ATC codes	x			X
DVA health services	Medical and allied health services in the Medicare Benefits Schedule (MBS) and extra items paid for by the DVA, delivered anywhere in Australia	DVA	July 2004–February 2011	MBS items DVA items	x			X
Admitted Patients Data Collection	Inpatient separations from all public and private hospitals in NSW	NSW MoH	July 2000–December 2011	ICD-10-AM	x	x	x	X
Emergency Department Data Collection	Emergency department visits to a subset of public hospitals in NSW	NSW MoH	January 2005–June 2012	ICD-9, ICD-10 SNOMED CT				X

*The program has ethical approval for annual data updates until 2015.

^Common years across all datasets are 2005–2009.

X denotes primary data sources; x denotes additional data sources.

*ICD-10* International Statistical Classification of Diseases and Related Health Problems 10th Revision.

*ICD-O-3* International Classification of Diseases for Oncology, 3rd Edition.

*ICD-9* International Statistical Classification of Diseases and Related Health Problems 9th Revision.

*ICD-10-AM* International Statistical Classification of Diseases and Related Health Problems, 10th Revision, Australian Modification.

*ATC* Anatomical Therapeutic Chemical.

*SNOMED CT* Systematized Nomenclature of Medicine—Clinical Terms.

#### Patient information

##### DVA client database (2004–2010)

Contains data on clients’ sex, dates of birth and death, level and history of health care entitlement (Gold, White, Orange) and postcode of residence history mapped to Statistical and Local Government Areas (SLAs [16] and LGAs [17]). The SLAs and LGAs form part of the Australian Standard Geographical Classification [18], established to enable the development of geography-specific statistics and classification of areas according to socioeconomic profile and remoteness. We did not obtain individual-level data on the service history of the clients in our cohort; however, almost all male DVA clients are veterans of the Australian armed forces and almost all female clients are dependants [14].

##### DVA residential aged care database (2005–2011)

The database contains data on clients’ admission and discharged dates in residential aged care, discharged reason and type of care received.

#### Registers

##### NSW Registry of Births, Deaths and Marriages (RBDM) (1994–2007)

Contains date and cause of death according to the Australian Bureau of Statistics (ABS) cause of death field (ICD-10-AM codes) [19].

##### NSW Central Cancer Registry (CCR) (1994–2009)

The CCR records cancer diagnoses reportable by law for NSW residents. The registry is operated according to the rules of the International Association of Cancer Registries [20] and records cancer type (ICD-O-3 and ICD-10-AM codes), date of diagnosis, degree of spread at the time of first diagnosis for solid tumours and the date and cause of death (cancer or non-cancer) [21].

#### Prescribed medicines and health service datasets

***Australian Government datasets***

##### Pharmaceutical Benefits Scheme and Repatriation Pharmaceutical Benefits Scheme (RPBS) (2004–2010)

DVA clients are entitled to all items listed on the Pharmaceutical Benefits Schedule (PBS-listed items) plus additional DVA-approved items (the combined listing hereafter referred to as the RPBS). DVA clients can also make *ad hoc* requests for the DVA to subsidize pharmaceutical items that are not RPBS-listed. The RPBS dataset contains information on all DVA-subsidized medicines including the item number, name and strength, date of prescription, date of supply, quantity supplied, number of repeats and benefit paid on all pharmaceutical items subsidized, in whole or part, by the DVA and dispensed in the community or in private hospitals. Patients are required to contribute to the cost of medicines via a co-payment. As of 1 January 2014, the co-payment is $AUD36.90 for general beneficiaries and $AUD6.00 for entitled DVA clients and concessional card holders (those covered by government entitlements such as low-income earners and welfare recipients).

Prior to 2012, the RPBS database only recorded pharmaceutical items attracting a government subsidy as the dispensing information for items costing less than the co-payment was not sent for reimbursement (as the individual has already paid the full cost). However, incomplete ascertainment of pharmaceutical items is not an issue for DVA clients or concession card holders, as all prescription medicines cost more than the concessional co-payment. In 2012, the government started collecting data on medicines under co-payment, so incomplete ascertainment of prescription medicines will no longer be an issue for the entire Australian population. One limitation of the database is that medicines prescribed to public hospital inpatients are generally not captured, as they are covered by the hospital budget. Further, the database does not capture over-the-counter medicines or prescription medicines not subsidized by the DVA.

##### DVA health services (2004–2011)

DVA clients are entitled to free or subsidized treatment from health care professionals including general practitioners, specialists, dentists and allied health professionals. They are also entitled to additional DVA-approved treatments not listed on the MBS for the general Australian population and DVA clients can make *ad hoc* requests for subsidy of non-listed items. This dataset contains the service item number, service category, date of service and amount paid for all in-hospital and out-of-hospital health services subsidized by the DVA, including physician visits, diagnostic and therapeutic procedures (including surgery), dental services, allied health services and pathology tests. While the Australian government sets MBS fees, patient co-payments are uncapped as health care professionals can charge any amount they choose for their services. However, if a health care professional elects to bill Medicare directly for a service that a patient receives (referred to as “bulk billing”), there is no patient co-payment. Under legislation, DVA clients are bulk-billed at the rates specified in the DVA health services schedules.

#### New South Wales Government datasets

##### NSW Admitted Patients Data Collection (APDC) (2000–2011)

This dataset is a census of all inpatient episodes from public, private and repatriation hospitals, private day procedures centres and public nursing homes in NSW. The APDC holds data on the dates of admission and separation, up to 50 diagnoses and procedures (ICD-10-AM), Australian Refined Diagnostic Related Groups (AR-DRGs), source of referral and separation mode (discharge, transfer or death).

##### Emergency Department Data Collection (EDDC) (2005–2012)

This dataset records all emergency department visits to 90 public emergency departments across NSW. It includes the date, reasons and outcome of the visit (e.g. admission to hospital, death or discharge). There are 150 emergency departments in NSW. Most of the larger departments contribute data to the EDDC so the dataset includes the majority of ED attendances [19]. Moreover, using the APDC, it is possible to identify ED presentations which resulted in an admission to hospital.

#### Data linkage

Data linkage was performed by the Centre for Health Record Linkage (CHeReL) [22], which maintains a linkage system for health-related data in NSW and the Australian Capital Territory in accordance with all ethical, legal, privacy and confidentiality requirements. The CHeReL keeps a Master Linkage Key that consists of contiously updated links between most NSW Health datasets.

The DVA provided the CHeReL with an encrypted client number and personal information for all clients residing in NSW. The CHeReL then assigned a project person number (PPN) to each DVA client. A “Project Key” containing the PPN and encrypted client number for each respective database was sent to the various data custodians, who decrypted the client number and attached the PPN and requested variables, which were then sent to the researchers stripped of identifying information such as name and address. The researchers joined all datasets using the PPNs.

### Ethics

Our cancer health services research program was approved by the NSW Population and Health Services Research Ethics Committee (approval number 2013/11/494) and the Department of Veterans’ Affairs Human Research Ethics Committee (E013/015). Our end-of-life research program will be conducted under the auspices of these approvals; however, research beyond 2015 will be subject to continued approvals.

### Study population

**Figure 1:**
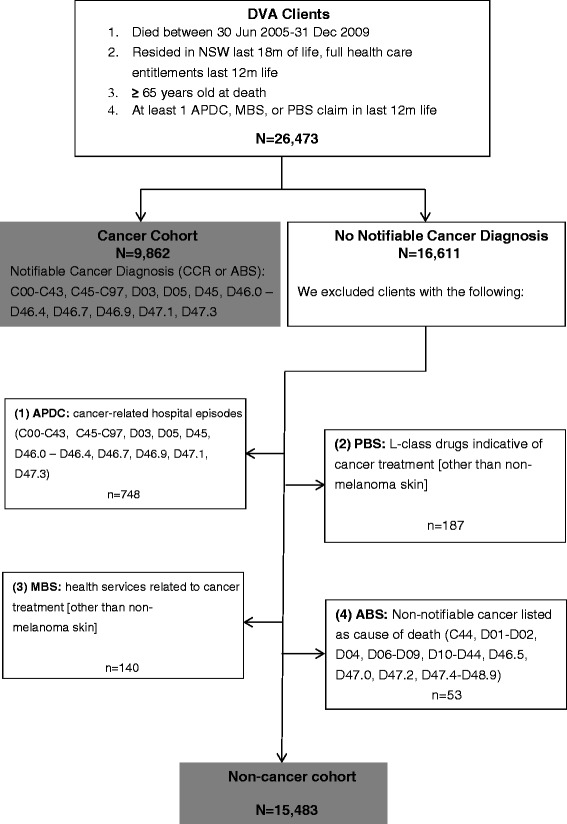
**Cohort inclusion criteria.**

We derived two cohorts for the purposes of our end-of-life research program; patients with evidence of a cancer diagnosis and those without a cancer history (see Figure 1). Both cohorts were restricted to DVA clients meeting the following criteria:

- Full health care entitlements for the last 12 months of life (to ensure complete capture of all health services and medicines).
- Resident of NSW for the last 18 months of life.
- ≥ 65 years old at death.
- Died between 30 June 2005–31 December 2009
- ≥ 1 service claim (hospital, health care service or medicine) in the last 12 months of life.

#### Cancer cohort eligibility criteria

We included decedents diagnosed with a notifiable cancer (C00–C43, C45–C97, D03, D05, D45, D46.0–D46.4, D46.7, D46.9, D47.1, D47.3) as recorded in the cancer registry (NSW CCR: January 1994–December 2008) or notifiable cancer recorded as any cause of death (RBDM: January 1994–December 2007).

#### Non-cancer cohort eligibility criteria

We included decedents meeting the following criteria:

1. No diagnosis of a notifiable cancer (C00–C43, C45–C97, D03, D05, D45, D46.0–D46.4, D46.7, D46.9, D47.1, D47.3) as reported in the cancer registry (NSW CCR: January 1994–December 2008).
2. Cancer not listed as any cause of death (RBDM: January 1994–December 2007).

We also applied additional criteria in order to account for the following: 1) death from cancer outside the dates for which we have cause of death information (i.e. after December 2007) or 2) receipt of a cancer diagnosis outside of NSW, in which case the information would not be captured in the NSW-based registry.

To account for the abovementioned scenarios and ensure our non-cancer decedent cohort did not include clients with a cancer diagnosis/cause of death, we screened all eligible non-cancer decedents for receipt of services or medications indicative of cancer treatment. Specifically, we developed a hierarchy based on hospital services, cancer drugs and other health care services:

1. Hospital Services (APDC): We excluded clients who received a cancer-related procedure(s) (C00–C43, C45–C97, D03, D05, D45, D46.0–D46.4, D46.7, D46.9, D47.1, D47.3) in hospital between July 2000 and December 2009.
2. Prescription drugs (R/PBS): We excluded clients who received cancer drugs between July 2004 and December 2009. We reviewed all L-class drugs (ATC, World Health Organization) to determine if they were indicative of cancer treatment; a clinical pharmacologist (NB) confirmed whether drugs administered individually or in combination with other L-class drugs reflected cancer treatment. Decedents who received L-class drugs were excluded from the non-cancer cohort except for the following: L01AA01, L01AA02, L01BA01, L01BB02, L01BC02 (in cream form only), L01XC02, L02AB02, L02AE03, L03AA02, L03AX03, L04AA06, L04AA13, L04AB01, L04AB02, L04AB04, L04AD01, L04AX01 and L04AX03. Clients who received intravenous fluorouracil (ATC code L01BC02 in intravenous form) were excluded from the cohort.
3. Health care services: We excluded clients who received medical services likely to indicate cancer surgery or treatment between July 2004 and December 2009. We reviewed all DVA health care services and excluded clients with a record of chemotherapy, radiation oncology, surgery or a multi-disciplinary cancer care/case conference. A practicing medical doctor reviewed the records of all patients in receipt of such services and confirmed they most likely reflected cancer care.
4. Finally, we excluded clients with a non-notifiable cancer cause of death as listed in the RBDM (ICD10: C44, D01–D02, D04, D06–D09, D10–D44, D46.5, D47.0, D47.2, D47.4–D48.9).

### Cause of death

We currently have the capacity to report cause of death for decedents who died between 2005 and 2007 as the ABS cause of death data are available in the NSW Registry of Births, Deaths and Marriages in this period. We utilized these data to determine the proportion of patients with a cancer diagnosis whose cause of death was also cancer and to understand the nature of other common medical conditions at the end of life in our cohort.

- We identified cancer causes of death using the codes C00-C43, C45–C97, D03, D05, D45, D46.0–D46.4, D46.7, D46.9, D47.1 and D47.3. All of these cancers are notifiable to the NSW CCR.
- We grouped non-cancer causes of death based on categories used by the Australian Institute of Health and Welfare (AIHW) and Charlson comorbidity index [23-26]. The most common non-cancer causes of death were heart failure (I21, I22, I25.2, I50, I70–I74, I79.0, R02, Z95.8, Z95.9), cerebrovascular disease (I60–I69), coronary heart disease (I20, I23, I24, I25.0, I25.1, I25.3–I25.9), chronic obstructive pulmonary disease (J40–J44) and dementia (F00–F03, F05.1, G30, G31). Deaths that did not fall into these categories were classified as “other”.

The NSW CCR also details cause of death in the period 1994–2008 categorized according to cancer type and non-cancer death, the latter not being specified beyond this category. As the ABS and NSW CCR registries both measure cause of death, we analysed clients with records in both registries (5,060) to determine the level of agreement. Overall, there was a 97.7% agreement between the ABS and NSW CCR for the underlying cause of death. The NSW CCR contains more specific information on cancer deaths than the ABS as it uses ICD-O-3 codes and pathology information, as such we will use this data source when examining differences in resource use and cost by cancer cause of death.

### Cohort characteristics

A total of 9,862 decedents met the eligibility criteria for our cancer cohort and 15,483 for our non-cancer cohort (Table 2). There were more males in the cancer cohort (68.4%) compared with the non-cancer cohort (51.4%), driven primarily by the high numbers of prostate cancer diagnoses. The median age of the cancer cohort was 86 years (range 65–107) and 87 years (range 65–111) in the non-cancer cohort; 52.9% of the cancer cohort and 59.6% of the non-cancer cohort died between 85 and 94 years of age. At the time of death, 61.9% of all decedents resided in major cities and 11.9% resided in the most socioeconomically disadvantaged neighbourhoods. Importantly, age at death, socioeconomic status and location of residence were very similar across both cohorts. Comorbidity burden, as measured by the Charlson index [23] was similar in both cohorts, however the percentage of decedents with a comorbidity burden of 3 or more was higher in the cancer cohort (17.4%) compared to the non-cancer cohort (10.2%). As a Charlson score is calculated using hospital admissions, it is likely to under-ascertain comorbidity. In contrast, the RxRisk [27] comorbidity score is based on prescriptions dispensed in the community, so more comorbidities are generally ascertained by this measure; 95% of both cohorts have at least one comorbidity based on RxRisk, and, the comorbidity burden does not differ between the two cohorts.

**Table 2:** **Characteristics of cancer and non-cancer cohorts**

	**Cancer cohort**	**Non-cancer cohort**
	***n*** **= 9,862**	***n*** **= 15,483**
	***n*** **(%)**	***n*** **(%)**
Sex		
Female	3,116 (31.6)	7,521 (48.9)
Male	6,746 (68.4)	7,962 (51.4)
Age at death		
65–74	294 (3.0)	254 (1.7)
75–84	4,075 (41.3)	5,028 (32.5)
85–94	5,215 (52.9)	9,232 (59.6)
95–104	277 (2.8)	958 (6.2)
≥105	1 (0.0)	11 (0.1)
Year of death		
2005	1,204 (12.2)	1,772 (11.4)
2006	2,236 (22.7)	3,199 (20.7)
2007	2,351 (23.8)	3,473 (22.4)
2008	2,133 (21.6)	3,619 (23.4)
2009	1,938 (19.7)	3,420 (22.1)
Location of residence at death (remoteness area)		
Major cities	6,147 (62.3)	9,530 (61.6)
Inner regional	2,777 (28.2)	4,400 (28.4)
Outer regional	872 (8.8)	1,410 (9.1)
Remote	39 (0.4)	81 (0.5)
Very remote	5 (0.1)	2 (0.0)
Missing	22 (0.2)	60 (0.4)
Socioeconomic disadvantage index at death		
(Most disadvantaged) 1–2	1,160 (11.8)	1,862 (12.0)
3–4	2,831 (28.7)	4,470 (28.9)
5–6	2,032 (20.6)	3,085 (19.9)
7–8	1,418 (14.4)	2,248 (14.5)
(Least disadvantaged) 9–10	2,019 (20.5)	3,183 (20.6)
Missing	402 (4.1)	635 (4.1)
Charlson comorbidity burden^1^		
0	3,105 (31.5)	4,451 (28.8)
1–2	1,500 (15.2)	2,068 (13.4)
≥3	1,713 (17.4)	1,578 (10.2)
No hospitalizations during calculation period (cannot calculate)	3,544 (35.9)	7,386 (47.7)
RxRisk comorbidity burden^2^		
0	461 (4.7)	846 (5.5)
1–2	1,298 (13.2)	2,130 (13.8)
3–5	3,865 (39.2)	6,104 (39.4)
≥6	4,238 (43.0)	6,403 (41.4)

^1^Calculated using hospitalizations in the 12-month period before the last 6 months of life (months 18 to 7 before death).

^2^Calculated using dispensing history in the 6-month period before the last 6 months of life (months 12 to 7 before death).

Of the 9,862 decedents in our cancer cohort, the most common cancer diagnoses were prostate (17.6%), colorectal (10.6%) and lung cancer (9.6%). 16.8% of decedents were diagnosed with more than one cancer (Table 3). Median age at diagnosis was similar across cancer types (80–85 years). However, there was substantial variation in the median time from diagnosis until death, ranging from 0.1 years in decedents with cancer of unknown primary site to 5.3 years for prostate cancer. The majority of cancers were *in-situ* or localized at the time of diagnosis but this varied according to cancer type. The degree of spread at diagnosis was unknown for many cancers.

**Table 3:** **Cancer cohort characteristics by cancer type**

	***n*** **(%)**	**Male (%)**	**Age at diagnosis**	**Time from diagnosis to death**	**Degree of cancer spread,** ***n*** **(%)**			
			**Median (range)**		**In-situ/localized**	**Regionalized**	**Metastatic**	Unknown*
				**Median (range)**				
All cancers	9,862 (100)	6,746 (68.4)	82.6 (54.5–105.6)	1.6 (0–15.8)	3,243 (32.9)	1,321 (13.4)	1,592 (16.1)	3,706 (37.6)
Prostate (C61)	1,735 (17.6)	1,735 (100)	80.8 (61.8–98.6)	5.3 (0–15.8)	586 (34.2)	53 (3.1)	150 (8.6)	943 (54.3)
Multiple cancers	1,652 (16.8)	1,322 (80.0)	83.5 (64.3–100.0)	0.9 (0–14.7)	548 (34.2)	217 (13.1)	300 (18.2)	578 (35.0)
Colorectal (C18–C21)	1,049 (10.6)	615 (58.6)	82.3 (56.8–101.7)	2.6 (0–15.7)	322 (31.7)	403 (38.4)	169 (16.1)	154 (14.7)
Lung (C33–C34)	951 (9.6)	630 (66.2)	82.6 (56.7–100.9)	0.4 (0–14.5)	202 (23.5)	131 (13.8)	294 (30.9)	311 (32.7)
Melanoma of skin (C43)	634 (6.4)	476 (78.1)	81.7 (57.8–99.3)	4.1 (0–15.8)	278 (42.7)	80 (12.6)	59 (9.3)	39 (6.2)
Haematological (C81–C96)	663 (6.7)	379 (57.2)	83.1 (60.8–102.4)	1.2 (0–14.8)	451 (72.2)	8 (1.2)	9 (1.4)	593 (89.4)
Other**	571 (5.80)	355 (62.2)	83.0 (60.8–100.3)	2.4 (0–15.1)	40 (6.4)	25 (4.4)	14 (2.4)	262 (45.9)
Breast (C50)	449 (4.6)	12 (2.7)	80.3 (54.5–105.6)	4.9 (0–15.2)	187 (41.6)	122 (27.2)	24 (5.4)	115 (25.6)
Digestive organs (C15–C17, C22–C24)	471 (4.8)	281 (59.7)	83.3 (59.8–97.1)	0.6 (0–15.7)	134 (31.6)	98 (20.8)	91 (19.3)	137 (29.1)
Unknown primary site (C26, C39, C76–C80)	389 (3.9)	209 (53.7)	85.5 (66.3–100.7)	0.1 (0–13.1)	1 (0.3)	13 (3.3)	242 (62.2)	132 (33.9)
Bladder (C67)	299 (3.0)	218 (72.9)	83.6 (64.0–98.3)	1.5 (0–15.6)	117 (40.9)	33 (11.4)	25 (8.4)	124 (41.5)
Pancreas (C25)	282 (2.9)	144 (51.1)	84.4 (67.9–97.9)	0.2 (0–7.8)	50 (20.4)	23 (8.2)	101 (35.8)	99 (35.1)
Head and neck (C00–C14, C30–C32)	226 (2.3)	176 (77.9)	81.6 (56.8–99.9)	2.9 (0–14.6)	96 (43.4)	49 (21.7)	7 (3.1)	73 (32.3)
Female genital organs (C51–C58)	183 (1.9)	0 (0)	82.3 (59.2–103.1)	1.2 (0–15.4)	52 (30.3)	38 (20.8)	54 (29.5)	39 (21.3)
Kidney (C64)	145 (1.5)	89 (61.4)	82.3 (63.3–94.9)	2.0 (0–13.6)	54 (40.6)	15 (10.3)	39 (26.9)	36 (24.8)
Connective and soft tissue (C45, C47–C49)	99 (1.0)	74 (74.7)	81.7 (64.6–95.0)	0.7 (0–13.7)	28 (29.5)	12 (12.1)	14 (14.1)	42 (42.4)
Brain (C71)	64 (0.6)	31 (48.4)	82.3 (54.6–93.4)	0.2 (0–10.6)	31 (51.7)	1 (1.6)	0	29 (45.3)

*Includes clients with cancer cause of death identified in death registry data; these clients do not have a cancer diagnosis in the NSW cancer registry (and no degree of cancer spread information).

**Includes C37–C39, C40–C41, C46, C60, C62, C63, C65, C66, C68, C69, C70, C72, C73–C75, D03, D05, D45, D46.0–D46.4, D46.7–D46.9, D47.1 and D47.3.

Information about cause of death is available for 13,575 decedents; 5,550 in the cancer cohort and 8,023 in the non-cancer cohort, all of whom died during the period 2005–2007 (Table 4). In the cancer cohort, the most common cause of death was cancer (58.9%), followed by heart failure (9.3%) and cerebrovascular disease (6%). In the cohort with no cancer diagnoses, the most common causes of death were heart failure (20.0%), cerebrovascular disease (15.4%) and coronary heart disease (12.8%).

**Table 4:** **Decedents in the cancer and non-cancer cohorts, by cause of death (died between 2005 and 2007)**

	**Cancer diagnosis (** ***n*** **= 5,550)**		**No cancer diagnosis (** ***n*** **= 8,023)**		**Total (** ***n*** **= 13,575)**	
	***n***	**%**	***n***	**%**	***n***	**%**
Cause of death						
Cancer (C00-C43, C45–C97, D03, D05, D45, D46.0–D46.4, D46.7, D46.9, D47.1, D47.3)	3,156	58.9	0	0	3,156	23.3
Heart failure (I21, I22, I25.2, I50, I70–I74, I79.0, R02, Z95.8, Z95.9)	515	9.3	1,603	20	2,118	15.6
Cerebrovascular disease (I60–I69)	333	6	1,232	15.4	1,565	11.5
Coronary heart disease (I20, I23, I24, I25.0, I25.1, I25.3–I25.9)	335	6	1,025	12.8	1,360	10
Chronic obstructive pulmonary disease (J40–J44)	175	3.1	545	6.8	720	5.3
Dementia (F00–F03, F05.1, G30, G31)	139	2.5	696	8.7	835	6.2
Other*	897	16.2	2,922	36.4	3,819	28.1

*Other ICD-10-coded causes of death not listed in the table.

### Outcomes of interest and statistical analyses

We will examine resource use, associated costs and quality of end-of-life care in the cancer and non-cancer cohorts. Most of the studies undertaken in this research program will focus on the last 6 months of life (the most common period of observation identified in our systematic review of end-of-life cancer care) [10]. For the purpose of our analyses, the last 6 months of life is defined as a period of 180 days including the day of death, based on constructed “months” that consist of 30 days each. However, we have the opportunity to study shorter (e.g. last month of life) or longer periods (last 12 months of life), also used previously in the literature. Additionally, we have the capacity to examine changes in outcomes as death approaches. In the first instance, we will report outcomes from January 2005 to December 2009, the period for which we currently have information about all health services, medicines dispensed and hospital and emergency department visits (Table 1).

Based on a 6-month end-of-life period, information is available for approximately 1 million health service records and 400,000 pharmaceutical claims in the cancer cohort. In addition, there are approximately 30,000 hospital separations and 12,000 emergency department admissions. As the non-cancer cohort is larger than the cancer cohort, we have more claims across all datasets and more person years of observation for this cohort (Table 5).

**Table 5:** **Number of records and person years in the last 6 months of life, by dataset, for cancer and non-cancer cohorts**

	**Total records**	**Records (last 6 months)**	**Total person years**
Cancer cohort (*n =* 9,862)			
Health services (MBS)	3,571,888	1,013,143	31,289
Prescription drugs (R/PBS)	2,142,162	407,200	31,289
Hospital services (APDC)	131,013	28,958	75,641
Emergency department (EDDC)	27,834	12,518	26,331
Non-cancer cohort (*n* = 15,483)			
Health services (MBS)	4,758,023	1,193,625	50,756
Prescription drugs (R/PBS)	3,459,325	594,474	50,756
Hospital services (APDC)	177,740	30,886	120,387
Emergency department (EDDC)	41,763	16,707	42,972

Below we describe the general approaches we will take to reporting end-of life resource use, costs and quality of end-of-life care. Our methods are informed by best practice approaches in the field. We have applied the Strengthening the Reporting of Observational studies in Epidemiology (STROBE) checklist [28] to this protocol (see Additional file 1) and will apply this checklist for all research generated from this program.

#### Resource utilization

We will use multiple metrics to examine the nature and extent of resource use at the end of life. For the cancer and non-cancer cohorts, we will report on resource use overall and by service type; at a patient level (e.g. description of services received by the typical decedent) and for each cohort. We will also stratify resource use by age, sex, cause of death, socioeconomic status and remoteness. We will examine the proportion of total resource use accounted for by each service (e.g. the proportion of total services accounted for by medications, hospitalizations). In the cancer cohort, we will also stratify resource use by cancer type and degree of spread (a proxy for cancer stage) at diagnosis. Each dataset will be used to highlight particular aspects of resource use.

##### Medicines use

We mapped pharmaceutical items to Anatomical Therapeutic Chemical (ATC) codes. We also developed a coding scheme to classify medicines according to their expected use towards the end of life. This scheme was developed based on expert input and published literature [29,30]. Medicines were classified based on whether prescriptions for each medicine are likely to remain unchanged, increase, decrease or both increase and decrease in the context of life-limiting illness. The categories are as follows:

- Symptom management (expect an increase towards the end of life)*-* such as analgesics for pain management, antiemetics or laxatives;
- Active treatment (use at the end of life may vary by patient and physician preference for active or palliative care)*-* drugs used for a limited time period with a specific purpose and measurable response such as cancer medicines, antifungal or antibiotic medicines to treat infections;
- Essential (expect prescribing to remain unchanged at the end of life)- ceasing the drug may potentially have serious adverse consequences for patients’ health/quality of life (e.g. anti-epileptic medications or diabetes medicines);
- Non-essential or preventative (expect a decrease towards the end of life in a terminally ill patient)- ceasing the drug would have limited impact on the quality of life of a terminally ill or palliative care patient (e.g. vitamins, statins).

##### Health care services

Due to the heterogeneous nature of the health service items, we have classified all MBS services and additional DVA-subsidized health services into the categories which will allow us to report in more detail the nature of health service use. This approach was taken as unlike other jurisdictions, Australian health care service items do not contain ICD codes, and their categorical structure does not lend itself to research on health service utilization. Our categories are as follows:

- Diagnostic tests, imaging and pathology;
- Visits to medical practitioners including general practitioners, specialists (e.g. psychiatry, emergency physician, pain and palliative care);
- Allied health (e.g. psychology, occupational therapy), nursing services and multi-disciplinary care plans and case conferences;
- Therapeutic procedures (e.g. chemotherapy, radiation oncology and nuclear medicine);
- Surgeries;
- Dental care;
- Items associated with the receipt of medical services that are for administration or billing purposes only and do not represent a health care service in isolation (e.g. management of bulk billing items that accompany physician attendances that are bulk-billed). These items will not be included in our resource use counts but will be included in our cost analysis.

##### Hospital admissions

We will examine the number of hospital separations and describe the nature and extent of hospital stays. An episode of care is defined as the period of admitted patient care between an admission and a separation, characterized by only one care type. This means that one hospital stay may include more than one episode of care (e.g. transition from acute care to a rehabilitation unit). In these cases, we can link multiple episodes at a patient level and report on the total length of stay and length of stay associated with each episode of care. Additionally, we will examine whether the hospital episode followed an emergency department presentation and the separation mode (e.g. death, discharge). Finally, we will report on the nature of hospital services provided (e.g. cancer-related, palliative care).

##### Emergency department visits

We will report on the number and outcome of visits including the number that result in hospital admission, death or discharge. ED presentations resulting in a hospital admission will also be identified from the APDC (source of referral = ED) given that the EDDC dataset does not capture all emergency departments in NSW. This will enable us to compare ED presentations rates resulting in a hospital admission in the APDC with those obtained directly from the EDDC dataset.

#### Costs

We will allocate unit costs to each item of resource use. We will report costs by service type at a patient level and for each cohort. We will also examine the contribution of each service type to total costs. Costs will be stratified by age, sex, cause of death, socioeconomic status, remoteness and, for the cancer cohort, cancer type and degree of spread at diagnosis.

Unit costs will be expressed according to a common financial year 2009/10. At the time of writing, the most current NSW Costs of Care Standards Report [31] is based on the financial year 2009/10, so unless otherwise stated, costs for all other datasets will also be benchmarked to that year.

##### Medicines costs

We will derive costs for individual items based on benefit paid and patient co-payment. The benefit paid includes dispensing fees, preparation fees, mark-ups and any other pharmacist fees. The benefit paid will then be inflated to a common price year, on a monthly basis (as PBS schedules are updated monthly), using inflation rates summarized in Table 6 derived from the AIHW published PBS pharmaceuticals index [32]. Where applicable, we will add the RPBS co-payment at the time of dispensing [33] to the benefit paid.

**Table 6:** **Derived inflation rates used in converting medicines costs and medical services costs to the June 2010 price level**

		**2004–05**	**2005–06**	**2006–07**	**2007–08**	**2008–09**	**2009–10**
Medicines costs^1^	Annual inflation rate (%)	0.1519	0.2123	0.2220	0.4832	0.1603	0.0200
	Monthly inflation rate (%)	0.0126	0.0177	0.0185	0.0402	0.0133	0.0017
Medical services costs (MBS)^2^	Annual inflation rate (%)	7.0593	4.7857	2.7389	2.3338	1.8166	
Medical services costs (dental)	Annual inflation rate (%)	5.6317	4.5525	4.5682	3.8390	4.1654	
Medical services costs (other)	Annual inflation rate (%)	3.5392	3.8714	1.1189	1.3020	5.6757	

^1^Derived inflation rates are applied on a monthly basis to reflect the fact that PBS price schedules are updated monthly. For example, if one AUD was spent in March 2005, then its inflated value in June 2010 can be calculated as 1 × (1 + 0.0126%)^3^ × (1 + 0.2123%) × (1 + 0.2220%) × (1 + 0.4832%) × (1 + 0.1603%) × (1 + 0.0200%) which equals 1.0114.

^2^Derived inflation rates are applied on an annual basis per November to October, to reflect the fact that the DVA Medical Fee Schedule is updated every November. For this reason, we adjusted the annual inflation rates for this sector so that they cover a year starting from November (the price index constructed by the Australian Institute of Health and Welfare is based on a financial year that starts from July). Based on these rates, if one AUD was spent on dental service in March 2005, then its inflated value in June 2010 can be calculated as 1 × (1 + 5.6317%) × (1 + 4.5525%) × (1 + 4.5682%) × (1 + 3.8390%) × (1 + 4.1654%) which equals 1.2491.

##### Health services costs

We will derive item costs for each service from the benefit paid and inflate costs to a common price year using inflation rates summarized in Table 6. For MBS, dental and allied heath items, we derived inflation rates from the AIHW Medicare medical services fees index, dental services price index and other health practitioner price index, respectively [32]. We will not make adjustments for co-payments, as all services provided to our cohort are bulk-billed.

##### Hospital costs

We will derive hospital admission costs, based on AR-DRG, length of stay and mode of separation, applying the approach described in Figure 1 of the NSW Costs of Care Standards Report 2009/10 [31]; using separate approaches for sub- and non-acute care (SNAP) patients, which include admissions like rehabilitation, palliative and maintenance care.

For SNAP AR-DRGs, we will apply *per diem* weights to the average SNAP cost ($11,582 in 2009/10) for each day in our observation period only (as admission may have occurred before the start of this period). SNAP trim points and cost weights for outlier days vary by class; however, a SNAP class is not supplied in our dataset. Consequently, we will use the inlier SNAP cost weight (0.0424) which is the same for all SNAP classes. This will be applied for each SNAP admission day in our observation period. Hospital costs for outpatients are assumed to be captured as MBS items.

For acute care admissions (non-SNAP AR-DRGs), the relevant cost weight will be multiplied by the base average cost ($3,840 excluding ED and ICU or $4,092 excluding ED only) with *per diem* rates applied for length of stay exceeding specified trim points up to 120 days for each AR-DRG. After a hospital stay of more than 120 days, a flat rate of $200 per day will be applied. Although the Costs of Care Standards cap the total cost at 365 days, to avoid underestimating costs of care, we will continue to apply a rate of $200 per day for hospitalization over 365 days. Where hospital episodes commenced prior to our observation period, we will only assign *per diem* costs to observed outlier days (days above the trim point); observed inlier days (below the trim point) are costed on a *pro rata* basis.

##### Emergency department costs

We will derive emergency department costs using the average cost of emergency presentations ($396/presentation in 2009/10). The NSW Costs of Care Standards Report 2009/10 (pp 14–15) assigns ED cost weights by “urgency and disposition group”, which is based on triage category, visit type and mode of separation [31]. However, triage category and visit type are not available in our dataset. Therefore, we will weight ED cost according to typical triage breakdown and visit type for each mode of separation from NSW Hospital Statistics [34].

##### Total cost

To calculate the total cost, we will adopt an approach to reduce the likelihood of double counting the cost of services when adding together health care services, medicines, hospital admissions and emergency department costs. We will assume any health services costs incurred at the same time as hospitalization will be captured by the AR-DRG cost, and therefore, we will only include out-of-hospital health services in our total cost calculation. We will count all RPBS items; however, for private hospital patients, we will deduct the pharmacy cost from the total AR-DRG cost when calculating total costs.

#### Quality of end-of-life cancer care: applying validated indicators to the Australian setting

There are a growing number of studies using validated indicators to assess quality and appropriateness of cancer care [35-38]; our recent systematic review identified 15 studies using “quality” end-of-life care indicators. These indicators were developed on the premise that quality end-of-life care involves withdrawal or reductions in life-extending (or “aggressive”) treatments and increased use of palliative services and hospice care. The first study reporting quality of care indicators based on administrative health data [36] covered multiple services such as administration of chemotherapy, emergency department visits, time spent in hospital and time spent in the ICU at the end of life. Some specific examples include the proportion of patients with at least one emergency department visit or hospital admission in the last month of life. These indicators have been influential, with many subsequent studies adopting similar indicators.

Across the 15 studies identified in our systematic review, there was some variability in the definitions of quality of care used; however, the aspects of resource use were similar, and most studies focused on services used in the last month or last 2 weeks of life. Commonly used “aggressive” indicators include intensive care or emergency department visits, inpatient hospital admissions, chemotherapy and life-sustaining treatments close to death. Palliative indicators focus on hospice care, pain relief (opioids) and primary and community care at the end of life.

To date, none of the indicators have been applied in the Australian setting. We will use best practice methods to adapt and refine these validated indicators of end-of-life cancer care using the datasets outlined in this protocol. To ensure our indicators are applicable to the Australian setting, we will consult with palliative experts (including clinicians and patients) and use our data on resource use in the last 6 months of life in our cancer cohort to empirically derive Australian-specific thresholds.

#### Factors associated with outcomes of interest

Regression methods to determine factors related to resource utilization and costs will be based on the specific question and the distribution of the data. For example, when modelling counts of services, we may use a Poisson or negative binomial analysis depending on whether the data are over-dispersed. Factors related to binary outcomes, such as quality of end-of-life care, will be determined using logistic regression. The literature suggests that a range of factors impact on the nature and extent of resource use at the end of life; we have the capacity to examine the impact of age [39-47], sex [39-47], comorbidity burden [40,48,49] and location of residence (geographical and residence in an aged care facility versus community dwelling decedents) [12,41] on outcomes of interest. We will also examine trends by year of death. Additionally, we will examine the impact of a number of clinical factors including cause of death (cancer versus non-cancer; where available) and, in the cancer cohort, cancer type, spread at diagnosis and time from diagnosis to death.

#### Dissemination plan

End-of-life care is a research priority in Australia and internationally. Research from this program will be submitted to international peer-reviewed journals, and results will be presented at national and international conferences. Particular journals and conferences will be focused on oncology, health services research, health economics and the implementation of health services research into clinical care and policy. We will consult clinicians, policy makers and consumers where appropriate for guidance in interpreting results and communicating results to target audiences; this may involve producing lay summaries of research findings. In accordance with our DVA data agreement, we will submit all data that will be communicated in the public domain to the DVA for review and approval. Direct access to the data and analytical files is not permitted without the expressed permission of the approving human research ethics committees and data custodians.

## Discussion

End-of-life care is a high-priority research area for health policy and planning. The topic has recently been the subject of editorials and research articles in the highest ranking general medicine and cancer journals internationally [2,3]. It has also been identified as a research priority by the leading Australian cancer advocacy group [50]. The research outlined in this protocol makes the best use of comprehensive linked datasets and addresses a key evidence gap in the Australian setting and internationally.

The large body of research on end-of-life patterns of care in the North American setting highlights the importance of the local environment and that the delivery of palliative care is highly jurisdiction specific [51]. The five papers reporting the results of research into end-of-life care in the Australian setting to date [7-9,11,12] have focused primarily on hospital services; our program will build on this work to provide a more comprehensive picture of end-of-life care. Additionally, this program is the first to apply quality of care markers of end-of-life care outside the North American setting.

The strengths of this program lie in the use of best practice methods to examine comprehensive data holdings including medicines, hospital services and other health care services. The limitations of using administrative data for research purposes are well recognized; however, as demonstrated in our recent systematic review, the volume of research using these data to examine the quality of end-of-life care continues to grow [10] and with this comes improved methodology such as validated indicators and risk-adjustment methods. While the DVA population represents a substantial proportion of the elderly population in Australia, the generalizability of our findings beyond DVA clients is a potential limitation of this program. Importantly, data linkage initiatives currently underway will improve data access and allow researchers to conduct observational research on end-of-life resource use and costs in the general adult population, building on the foundations of this program.

## Additional file

Additional file 1:
**STROBE Statement checklist.** Checklist of items that should be included in reports of *cohort studies*.

## Abbreviations

ABS: Australian Bureau of Statistics
APDC: Admitted Patients Data Collection
CCR: Central Cancer Registry
CHeReL: Centre for Health Record Linkage
DVA: Department of Veterans’ Affairs
EDDC: Emergency Department Data Collection
ICD: International Classification of Diseases
MBS: Medicare Benefits Schedule
NSW: New South Wales
PBS: Pharmaceutical Benefits Scheme
RBDM: Registry of Births, Deaths and Marriages
RPBS: Repatriation Pharmaceutical Benefits Scheme
